# Peripheral Blood and Salivary Biomarkers of Blood–Brain Barrier Permeability and Neuronal Damage: Clinical and Applied Concepts

**DOI:** 10.3389/fneur.2020.577312

**Published:** 2021-02-04

**Authors:** Damir Janigro, Damian M. Bailey, Sylvain Lehmann, Jerome Badaut, Robin O'Flynn, Christophe Hirtz, Nicola Marchi

**Affiliations:** ^1^Department of Physiology Case Western Reserve University, Cleveland, OH, United States; ^2^FloTBI Inc., Cleveland, OH, United States; ^3^Neurovascular Research Laboratory, Faculty of Life Sciences and Education, University of South Wales, Wales, United Kingdom; ^4^IRMB, INM, UFR Odontology, University Montpellier, INSERM, CHU Montpellier, CNRS, Montpellier, France; ^5^Brain Molecular Imaging Lab, CNRS UMR 5287, INCIA, University of Bordeaux, Bordeaux, France; ^6^Cerebrovascular and Glia Research, Department of Neuroscience, Institute of Functional Genomics (UMR 5203 CNRS—U 1191 INSERM, University of Montpellier), Montpellier, France

**Keywords:** neurovascular unit, blood biomarkers, saliva, concussion, epilepsy, neurodegeneration, traumatic brain injury, extreme sports

## Abstract

Within the neurovascular unit (NVU), the blood–brain barrier (BBB) operates as a key cerebrovascular interface, dynamically insulating the brain parenchyma from peripheral blood and compartments. Increased BBB permeability is clinically relevant for at least two reasons: it actively participates to the etiology of central nervous system (CNS) diseases, and it enables the diagnosis of neurological disorders based on the detection of CNS molecules in peripheral body fluids. In pathological conditions, a suite of glial, neuronal, and pericyte biomarkers can exit the brain reaching the peripheral blood and, after a process of filtration, may also appear in saliva or urine according to varying temporal trajectories. Here, we specifically examine the evidence in favor of or against the use of protein biomarkers of NVU damage and BBB permeability in traumatic head injury, including sport (sub)concussive impacts, seizure disorders, and neurodegenerative processes such as Alzheimer's disease. We further extend this analysis by focusing on the correlates of human extreme physiology applied to the NVU and its biomarkers. To this end, we report NVU changes after prolonged exercise, freediving, and gravitational stress, focusing on the presence of peripheral biomarkers in these conditions. The development of a biomarker toolkit will enable minimally invasive routines for the assessment of brain health in a broad spectrum of clinical, emergency, and sport settings.

## Introduction: From Blood–Brain Barrier to Blood–Brain Dynamic Interface

**Figure 1:**
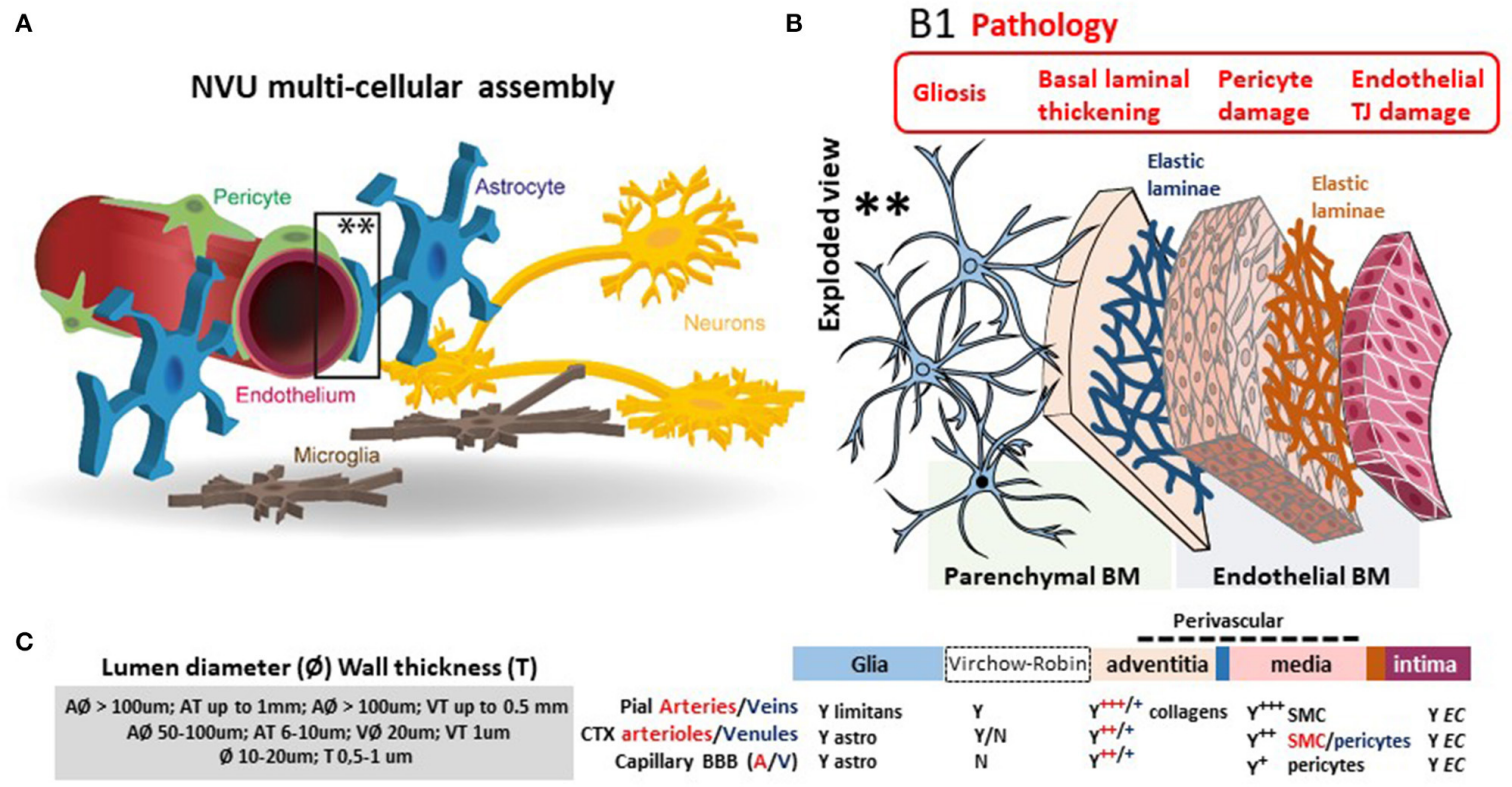
The dynamic NVU multicellular layout. (**A**) Within the NVU, the BBB encapsulates a set of unique properties of the microvascular capillary and post-capillary venules. The BBB endothelium lacks fenestrations, is assembled by structured tight junctions (TJs), and expresses luminal or abluminal transporters, altogether finely regulating brain homeostasis for proper neuronal physiology. These endothelial specializations are generated and controlled by precise interactions with pericytes, astrocyte end-feet, and microglial cells, all participating to the NVU. **(B)** Exploded view to illustrate the varying cellular composition and wall thickness of the intima, media, and adventitia layers. The endothelial basement membrane (BM) embeds pericytes. A second basement membrane is deposited by astrocytes and surrounds the end-feet. At the capillary level, the endothelial and parenchymal basement membranes merge. At the post-capillary venules, the two basement membranes separate to provide a perivascular space that allows for immune cells homing. (**B1**) Commonly reported pathological modifications leading to BBB permeability or NVU damage. **(C)** The cerebrovasculature in numbers (A, arteries; V, veins; CTX, cortex; SMC, smooth muscles cells). Proper BBB commences as the deepening cortical arteries (diameter > 100 μm in mice) branch into arterioles (diameter 15–50 μm; wall thickness 5–10 μm) and capillaries (diameter <10 μm; wall thickness a few μm). Pial vessels have glia *limitans* and an anatomically distinguishable Virchow-Robin space **(B)**. Blood flow velocity rates in cortical mouse arterioles and capillaries are 3 and <0.5 mm/s, respectively. Capillary blood flow decreases with cortical depth (down to 0.1 mm/s). Diameter ranges (rodent) and anatomical abundance of glia, Virchow-Robin space, collagens, and mural cells (smooth muscles or pericytes) is provided. The multicellular layering within the tunica media (sign +++ indicates more than three smooth muscle cells at the arteries; + indicates one layer of pericytes at the BBB) is the major determinant of vascular thickness and elasticity. Original images by NM and IGF graphical service.

**Figure 2:**
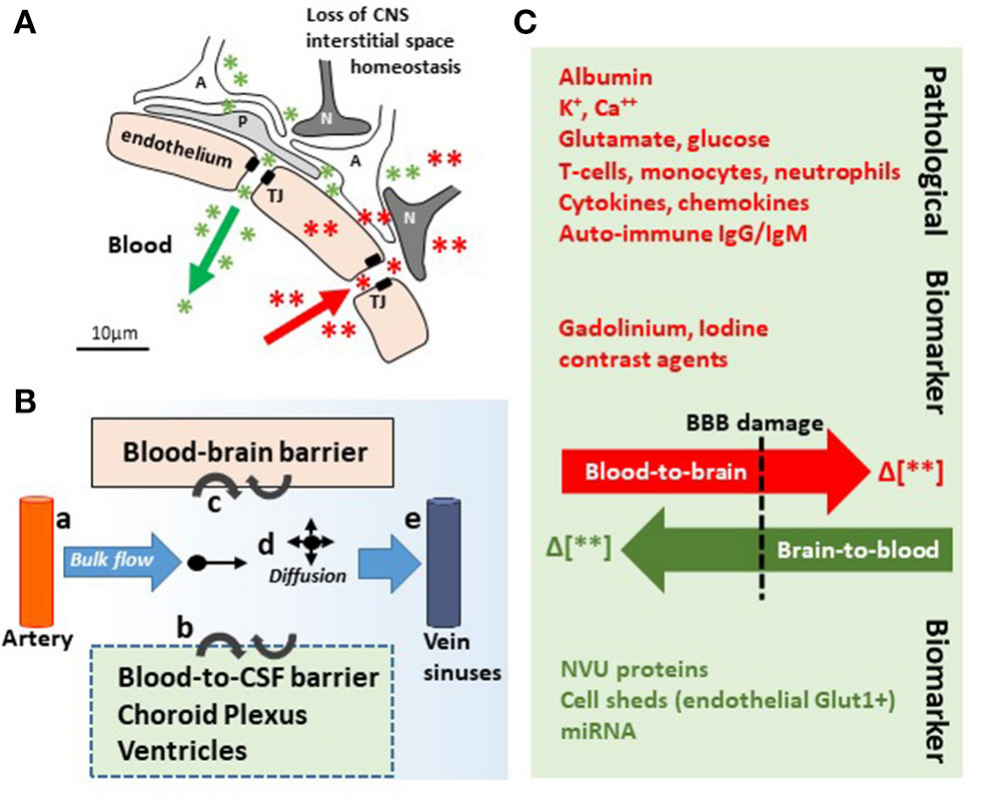
In and out the brain: plausible biomarker exit routes and transport. **(A)** NVU cell disassembly causes disruption of brain homeostasis, allowing blood (red asterisks and arrow) and brain (green asterisks and arrow) components directly crossing the permeable BBB (TJ, tight junctions; A, astrocytes; P, pericytes; N, neurons). **(B)** Brain fluid paths regulate the movement of molecules (e.g., biomarkers) within the brain parenchyma into the blood and the CSF: (a) the CSF is produced by subarachnoid arteries and (b) the choroid plexus (blood-to-CSF barrier). By bulk flow mechanisms, the CSF diffuses at the cortical levels and in periventricular organs, constituting a possible vehicle for biomarker transport. (c) BBB damage allows biomarkers exiting (or entering) the brain. (d,e) Parenchymal bulk flow and CSF reabsorption occurs at larger veins, dural venous sinuses, and dural lymphatic vessels, sites where biomarkers could accumulate. **(C)** Concentration gradients (green/red Δ or arrows) between the peripheral blood and the brain parenchyma are the underpinning for pathological modifications and the driving force for biomarkers (e.g., MR contrast agent brain entry, red; protein biomarkers brain exit, green). Original images by NM.

**Figure 3:**
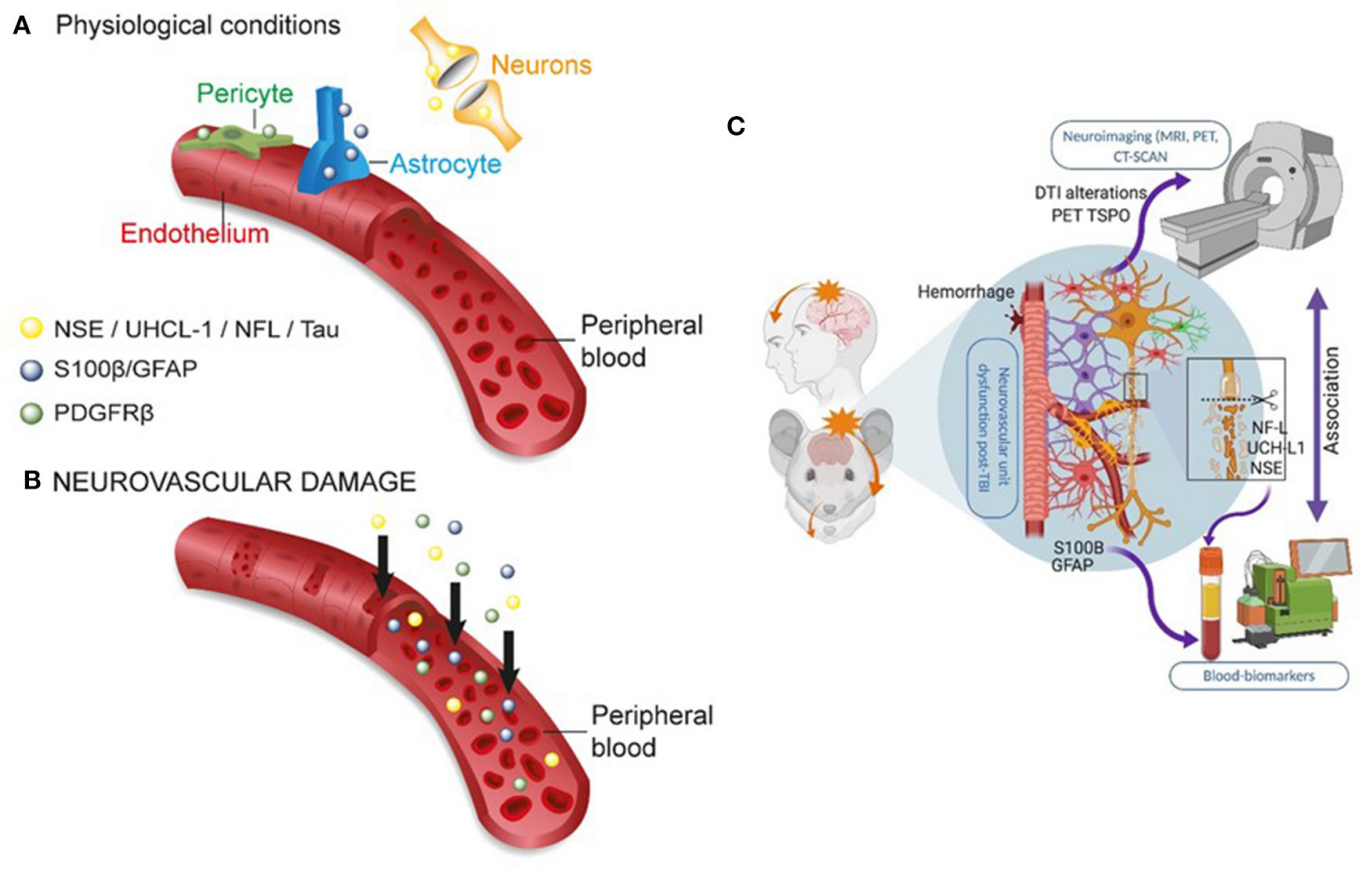
Peripheral biomarkers of BBB permeability and brain damage. **(A)** Under physiological conditions, BBB tightness within a healthy NVU limits proteins from exiting the brain into the peripheral blood. **(B)** In conditions of brain damage, each neurovascular cell acts as a source of specific biomarker(s) (color coded), accessing the peripheral blood across a leaky BBB. The production or secretion of biomarkers at each NVU cell type depends on the severity, time, and the progression of disease states (see Table 1). **(C)** Integrating the use of brain imaging and peripheral biomarkers is a developing strategy to detect brain damage and to validate the usefulness of specific biomarker proteins in peripheral fluids. Original images by NM, JB, and IGF graphical service.

The blood–brain barrier (BBB) is the complex and finely tuned network of brain capillaries governing the homeostatic exchange of ions, molecules, and cells between the brain and the peripheral blood (1–3). The importance of the BBB in the understanding and diagnosis of neurological disorders and brain health is recognized (4). The notion of BBB has evolved from that of a static brain shield to that of a dynamic blood–brain interface where endothelial cells continuously communicate with mural cells (pericytes and smooth muscle) and glia (astrocytes and microglia), located near neurons and spatially assembled to constitute the neurovascular unit (NVU) (Figure 1) (2). A precise layering of cells and extracellular matrixes forms an impermeable wall (Figure 1B). BBB dysfunction has etiologic and diagnostic significance (4), and BBB permeability is a key element of perivascular and neuroinflammation (Figure 1B1) (5, 6). Increased BBB permeability provokes an immediate loss of homeostatic control of ions, ATP, and neurotransmitters levels in the brain, promoting abnormal synaptic transmission or neuronal firing, possibly leading to neurological sequelae (6–13). On the other hand, neuronal activity significantly influences cerebrovascular functions in health and disease conditions (14, 15). Diagnostically and because of increased BBB permeability, peripherally injected imaging contrast agents can access the brain parenchyma while a suite of central nervous system (CNS) proteins (see Table 1) or nucleic acids [circulating free DNA and microRNA; for a review see (44, 45)] can exit into the peripheral blood (Figures 2A–C, 3A–C). Contrast MRI and CT scans are common clinical tools, while monitoring the levels of CNS proteins in peripheral body fluids represents a novel strategy for identifying BBB and neuronal damage (46). Importantly, the NVU connects with specialized brain acellular spaces through which the cerebrospinal and interstitial fluids carry ions, molecules, and proteins across the parenchyma or toward waste clearance pathways (Figure 2B) (47–50). This spatial perivascular and interstitial connectivity is important in the context of contrast-based brain imaging, possibly influencing the availability of biomarkers and their exit trajectories from the CNS [Figure 2; see (46, 51, 52) for a review]. Starting from these fundamental concepts, we here examine the evidence supporting the development and the use of specific peripheral biomarker proteins to detect glioneuronal damage and BBB permeability in a plethora of clinical, emergency and sport-related settings.

**Table 1:** Peripheral biomarker proteins of glio-neuronal damage and BBB permeability.

**Proteins**	**MW (kDa)**	**Role as biomarker**	**Estimated half-life in blood**	**Usage temporal trajectories**	**Source**	**Sampling methods**	**CNS disease**	**Reported (and varying) blood baselines**	**References**
GFAP	50	Astrocyte damage or astrogliosis	48 h (16)	Acute and Subacute (hours–days) (17)	Astrocyte cytoskeleton No clearly reported extra-cranial sources (18)	Venipuncture CSF	TBI Multiple sclerosis AD	Baseline 0.01 ng/ml TBI with negative CT 0.21 ng/ml TBI with positive CT 0.73 ng/ml	(17–21)
S100B	11	BBB and astrocyte damage, astrogliosis	2–6 h (22)	Acute (min, hours) (17)	Astrocyte calcium binding protein CNS development Extra-cranial sources [adipocytes (18)]	Venipuncture CSF Urine Saliva	TBI Epilepsy Multiple sclerosis	Pediatric 0.11 ng/ml (23) Adults 0.045 ng/ml (24) (sub)concussion, mTBI: 0.1 ng/ml (25, 26)	(17, 23, 25, 27–29)
UCH-L1	24	Neuronal cell damage (30)	7–9 h (31)	Acute (min, hours)Subacute (days) (17)	Axonal integrity Extra-cranial sources [neuromuscular junction (18)]	Venipuncture CSF	TBI Neurodegeneration	Pediatric 0.09 ng/ml (19) TBI with negative CT: 0.14 ng/ml TBI with positive CT: 0.44 ng/ml TBI with negative CT 261 pg/ml (21)	(19, 21)
NSE	47	Neuronal cell damage	30 h (32)	Acute (min, hours)Subacute (days) (17)	Neuron cytoplasmic enolase Detected in blood erythrocytes	Venipuncture CSF	TBI Epilepsy	Adults 6.1 μg/ml (24)	(33, 34)
NFL	68	Axonal injury, neuronal death	3 weeks (35)	Subacute days to weeks (17) and chronic	Neuron class IV intermediate filaments of cytoskeleton	Venipuncture CSF	TBI Neurodegeneration Multiple sclerosis	Threshold CSF 386 ng/ml (36)	(37, 38)
PDGFRβ	123	Pericyte reactivity or damage	na	Subacute (39)	Pericytes–endothelial interface	CSF	Neurodegeneration	See (40) for graphic baseline (115 % increase in CSF between no and mild cognitive impairment)	(39)
Tau, pTau	50–80	Neuronal or axonal damages, neurodegeneration	10 h (41)	S majuscle, subacute, and chronic (17)	Neuronal microtubule-associated proteins Aggregates into neurofibrillary tangles	Venipuncture CSF	Neurodegeneration AD	Blood total and phosphorylated tau, (42) T-tau control = 65.59 fg/ml P-tau control = 20.85 fg/ml P-tau/T-tau ratio control = 30.94 Total-tau in serum 4.4 pg/ml (24) Threshold CSF P-tau 78 pg/ml (39)	(17, 24, 43)

## Peripheral Biomarkers: Basic Concepts and Focus on Traumatic Brain Injury

Elevated BBB permeability, or dysfunction, occurs in response to an acute injury (e.g., head trauma, stroke, and status epilepticus) and may be present throughout CNS disease progression (e.g., neurodegeneration, epileptogenesis, and multiple sclerosis), often due to inflammation (5–7, 13). Peripheral biological fluids represent suitable matrices to detect and quantify brain-derived proteins reporting BBB permeability and susceptibility to glio-neuronal damage (27–29, 53). Table 1 provides a list of protein biomarkers and their characteristics, properties, and proposed use in diagnostics. In general, peripheral biomarker proteins must (i) be present in brain interstitial fluids or be released by neurovascular cells into the interstitial or perivascular spaces, reaching the peripheral blood across a leaky BBB or by cerebrospinal fluid (CSF)–blood exchange (Figures 2A, 3A,B); (ii) have a concentration gradient driving passive diffusion [Figure 2C; see (29)]; (iii) have a known and appropriate half-life to allow diagnostic interpretation (29) (biomarker half-life in peripheral fluids may impact usefulness in acute vs. long-term settings; see Table 1); and (iv) have a low molecular weight to allow a rapid egress across the damaged barriers or interfaces (19, 25, 27).

The bulk of neurological clinical biomarker literature has often focused on traumatic brain injury (TBI), with a recent emphasis on mild TBI (mTBI) (18, 54). Within this framework, the astrocytic protein S100B (55) has been examined as a peripheral biomarker of BBB permeability and gliosis (Table 1 and Figures 3A,B). Early proof-of-principle studies showed serum S100B levels to rapidly increase in response to a sudden BBB permeability, supporting the hypothesis that perivascular S100B can readily exit the brain (27, 28, 56). S100B was reported to rule out mTBI sequelae in emergency room settings (57), and measurement of blood S100B levels displayed a 99.7% negative predictive value (NPV) (57–60). Further evidence indicated that monitoring S100B after a mTBI could override the need for a CT scan for the identification of intracranial injury, with an excellent NPV (61). However, another study reported no relationship between serum S100B concentration and mTBI severity (62). In sports, S100B blood levels increased immediately after football games as compared to pregame baselines in players experiencing repeated head hits (25, 63). The evidence of a rapid S100B surge in blood after sub concussive hits was confirmed in follow-up studies (63–65). Importantly, extra-CNS sources of S100B were reported, representing a potential confounding factor if timing of blood draws in relation to injury is not adequately controlled and standardized (18, 66). These concerns have been discussed in (23, 67, 68).

The astrocytic glial fibrillary actin protein (GFAP) and the neuronal ubiquitin carboxyl-terminal hydrolase isoenzyme L1 (UCH-L1) are important biomarker candidates for glioneuronal damage (Table 1 and Figures 3A,B). UCH-L1 is also expressed at the neuromuscular junction (69, 70) while the contribution of extracranial sources of GFAP is debated (20, 71, 72). Monitoring of blood GFAP and UCH-L1 levels was used to grade brain injury after TBI. GFAP and UCH-L1 levels were increased in non-concussive and concussive head trauma as compared to body trauma (73, 74). The analysis of blood GFAP (or S100B) levels within 24 h from the head injury was proposed as a means to improve the detection of TBI and to identify patients in need of a subsequent MRI, in addition to routine CT surveillance (75, 76). GFAP and UCH-L1 blood levels were used to rule out intracranial injuries and the need for CT scans, showing high test sensitivity and NPV (21). One study reported no significant difference in blood UCH-L1 between control and players who sustained repetitive head hits (77). Collectively, this evidence points to GFAP as a diagnostic candidate to be used in TBI (33, 54, 71, 72). In two studies (78, 79), however, GFAP and UCH-L1 levels were below the lower limits of quantification or detection (LLOQ or LLOD, respectively) in a percentage of both TBI and trauma control groups, representing a possible concern for estimating NPV (20, 80).

Important biomarkers detecting neuronal damage are myelin basic protein (MBP), neuron-specific enolase (NSE), tau, and neurofilament light chain [NfL; Table 1 and Figures 3A,B; see (43)]. Blood MBP levels were unchanged in a pediatric mTBI population as compared to controls. Interestingly, MBP levels remained elevated for up to 2 weeks in case of intracranial hemorrhage (81). NfLs are found in axons and have been proposed as biomarkers of axonal damage triggered by mTBI, for example, after an amateur boxing bout (82–84). S100B levels were also increased following amateur boxing (85). Further evidence indicated neurofilament heavy chain increase after mTBI (82). Finally, NSE levels in CSF were shown to be proportional to TBI severity, in the setting of moderate or severe TBI (86–88). NSE in the blood is less investigated due to its presence in erythrocytes (89, 90). Collectively, these data support the further development of blood biomarker toolkits of TBI, with a special relevance to mild head injury and sport-related (sub)concussions, when emergency and sideline diagnostic solutions need to be readily accessible.

## Phosphorylated TAU as an Emerging Blood Biomarker of Alzheimer's and Neurodegenerative Diseases

Accumulating evidence points to blood phosphorylated tau as a promising biomarker to improve the diagnosis and staging of and to enable trials in Alzheimer's disease (AD) subjects. In a cross-sectional study performed in AD patients, phosphorylated tau isoforms were used as diagnostic biomarkers to track disease progression (91). A method measuring attomolar concentrations of tau isoforms in plasma was implemented using stable isotope labeling kinetics and mass spectroscopy. Changes in plasma p-tau, particularly p-tau217, mirrored specific changes in CSF to detect phosphorylation of soluble tau and amyloidosis. No correlation was found between CSF and plasma p-tau202 levels. Plasma p-tau217 level distinguished amyloid-negative from amyloid-positive groups regardless of the cognitive status, indicating that p-tau217 in plasma may be an accurate biomarker of abnormal brain tau metabolism. Furthermore, a longitudinal study of familial AD (presenting pathogenic mutations in PSEN1 or APP genes) included 19 symptomatic and 51 asymptomatic participants where plasma p-tau181 levels were quantified by using a single-molecule array (Simoa) method (92). Elevated plasma p-tau181 concentrations segregated symptomatic mutation carriers from non-carriers. In another cross-sectional study including the Arizona-based neuropathology cohort (37 AD and 47 without AD), the Swedish BioFINDER 2 cohort [121 AD, 178 mild cognitive impairment [MCI], 301 without AD, and 99 other neurological disorders], and a Columbian autosomal-dominant AD kindred (365 PSEN1 E280A mutation carriers and 257 mutation non-carriers), plasma tau phosphorylated at the threonine 217 (p-tau217) was quantified by the Meso Scale Discovery (MSD) assay as a diagnostic AD biomarker (93). Among 1,402 participants from the three cohorts, plasma p-tau217 discriminated AD from other neurological disorders with higher accuracy compared with plasma p-tau181, plasma Nfl, CSF p-tau181, and CSF Aβ42:Aβ40 ratio. A positive correlation between CSF and plasma p-tau217 was found in the Swedish BioFINDER 2 cohort. Finally, a high-sensitivity immunoassay measuring p-tau181 in plasma and serum was developed (94). A positive correlation was reported between plasma and CSF p-tau181 levels, distinguishing Aβ-negative cognitively unimpaired older adults from Aβ-positive older adults and Aβ-positive individuals with MCI.

Furthermore, at the BBB, the low-density receptor-related protein 1 (LRP1) plays an important role in regulating cerebrovascular permeability (95). sLRP1, a truncated soluble form of LRP1, freely circulates in plasma, and it sequesters unbound Aβ in the peripheral circulation (96). Plasma sLRP1 levels are significantly reduced in AD patients, and sLRP1 binding to Aβ is disrupted by oxidation (96, 97). Impaired sLRP1-mediated binding of plasma Aβ was suggested as an early biomarker for MCI preceding AD-type dementia (97). In summary, this evidence supports the further development of tau-based blood biomarkers as an accessible test for the screening and diagnosis of AD within the spectrum of cognitive impairments and dementia.

## Peripheral Biomarkers of BBB Permeability and Seizure Conditions

The use of blood biomarkers extends to epilepsies, a cluster of diseases where BBB damage represents an etiological or a contributing pathophysiological player (98–100). A first study (67) demonstrated that blood S100B is elevated at seizure onset and after seizures, in support of the hypothesis that BBB damage may trigger a seizure (7, 101–103). A systematic review analyzed 18 studies and a total of 1,057 subjects, indicating that epileptic patients displayed elevated S100B blood levels as compared to controls (104). Meta-regression analyses showed that gender and mean age can impact serum S100B levels (104). Another study correlated MRI T1 peri-ictal imaging to blood S100B in drug-resistant epileptic patients, confirming the increase in BBB permeability during a seizure (105). Increased S100B blood levels were reported in pediatric temporal lobe epilepsy, with blood samples obtained 30 min after a complex partial seizure (106).

Children suffering from intractable focal epilepsy displayed elevated blood S100B levels as compared to controls (107). One study included 39 patients suffering from simple febrile seizures and age- and sex-matched controls, showing no S100B differences between groups when assessed immediately after seizures (108). These findings were corroborated in a follow-up study, (109) with the conclusion that febrile seizures are relatively harmless to the developing brain. Currently, a clinical trial is investigating whether S100B, as well as other protein biomarkers, increase in blood after a first generalized seizure could be used to predict first-to-chronic seizure conversion in adult subjects (https://clinicaltrials.gov/ct2/show/NCT02424123). Moreover, NSE elevations were reported in blood over time in patients affected by temporal lobe and extratemporal lobe epilepsies (110). Finally, recent evidence indicates miRNA in blood, or body fluids, as potential biomarkers indicating neurovascular and neuroinflammatory modifications occurring in specific forms of epilepsies [see (111–113) for comprehensive topic reviews]. In summary, blood biomarkers could represent a surrogate method of clinical electroencephalographic explorations to examine damage and brain neurophysiology in epileptic patients.

## Imaging BBB Permeability and Brain Damage: Is the Integration With Blood Biomarkers Possible?

Available evidence supports the prospective use of blood biomarkers to detect NVU damage in acute and chronic neurological conditions. In this context, can peripheral biomarkers replace brain imaging? This is an important question especially if one considers the logistics (scarce imaging availability in rural areas and emergency, sport, and combat settings) and economic advantages that come with peripheral biomarkers, notwithstanding the complications associated with radiation exposure (e.g., CT scan). As a result, the diagnostic equivalence of blood biomarkers and enhanced MRI or CT scans (114–119) is being investigated. Accumulating evidence has shown that mTBI represents an optimal clinical arena to study the usefulness of imaging and peripheral biomarkers, also fulfilling an urgent clinical need (120–122). Neuroimaging techniques [CT scan (61)] show limitations for the diagnosis of mTBI patients (122, 123). Importantly, blood levels of GFAP, tau, and NfL were higher in patients with TBI-related findings on CT as compared to subjects presenting with normal CT, where the only significant predictor of damage was GFAP (124). Combining the biomarkers tau, NfL, and GFAP showed a good discriminatory power for detecting MRI abnormalities, even in mTBI patients with a normal CT (124). Furthermore, peak serum S100B levels negatively correlated with resting-state brain connectivity and behavioral outcomes in mTBI to severe TBI cases (125). S100B has proven its high NPV to rule out intracranial bleeding in patients after mTBI. However, its specificity for brain parenchyma structural lesions remains debated, and MRI is required for a specific explanation of clinical symptoms (76, 126, 127). Positron emission tomography (PET) and radiolabeled biomarkers were tested along with blood biomarkers. The [^18^F]AV1451 (flortaucipir) tau ligand was detected at the white/gray matter junction in frontal, parietal, and temporal brain regions, a typical localization of chronic traumatic encephalopathy (CTE) and tauopathy in veterans. Elevated levels of Nfl were also reported in plasma (43). Finally, TBI is associated with inflammation as blood levels of IL6, TNFα, and VEGF were increased in CT- and MRI-positive patients as compared to controls (126).

Importantly, newer brain imaging approaches are being tested. Proton magnetic resonance spectroscopy (^1^H-MRS) represents an emerging neuroimaging modality to track the metabolic changes occurring after TBI (128, 129). Spectroscopy can predict changes of key metabolites such *N*-acetylaspartate (NAA), a marker of neuronal loss (130), and its early decrease associates with long-term poor outcomes in clinical pediatric mTBI and moderate TBI (130). Experimentally, spectroscopy modifications post injury were linked to altered astrocyte metabolism (131). Brain structural changes observed using diffusion tensor imaging were correlated to astrocyte dysfunction and astrogliosis at early (1–7 days) and late (60 days) time points after injury (132, 133). Tractography provides an opportunity for measuring structural alterations in the white matter that are not detected by conventional structural MRI (134). Magnetic encephalography has also been proposed to study mTBI damage, in addition to being used for post-traumatic stress disorders (135, 136). Collectively, these data underscore the need for integrating the temporal and quantitative profiles of emerging imaging read-outs with the dynamics of peripheral biomarker of NVU damage. These studies will allow us to fully understand whether blood biomarkers can reliably act as surrogates for brain imaging.

## Saliva as a Biomarker Matrix: General Concepts

**Figure 4:**
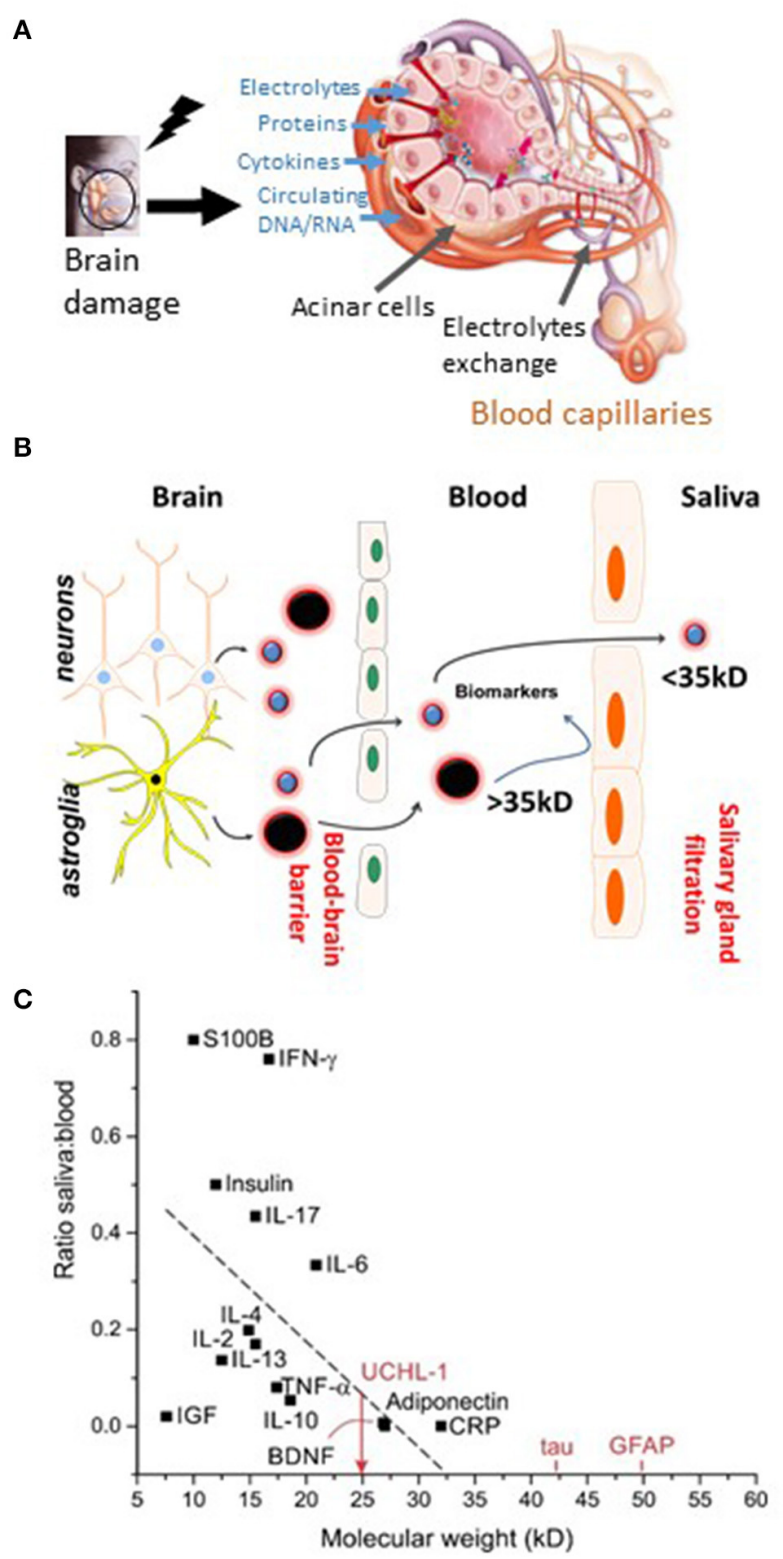
The blood-to-saliva interface and proposed salivary biomarkers. **(A)** Schematic representation of molecular transport or passage from blood into salivary glands. Salivary glands are highly vascularized, enabling exchange of blood-based constituents (ions, proteins, etc.). Alterations in the molecular composition of the blood may lead to modifications of the composition of saliva. **(B)** Biomarker extravasation from brain to blood depends on the permeability of the BBB to a given biomarker. Under normal conditions and when the BBB is intact, endothelial tight junctions restrict the passage of polar or large (>~300 Da) molecules. When the BBB is breached, appearance in the blood of brain-derived protein biomarkers occur. Next, the passage of protein from blood to saliva is proposed. **(C)** Possible protein ratio of saliva to blood for biomarkers and pro-inflammatory factors (see text for details and references). Original images by DJ and CH.

Another key cellular “barrier” can be exploited for diagnostic purposes, namely, the salivary glands and gingival vessels, both interfacing with the peripheral blood (Figures 4A,B) (53, 137–144). While plasma and serum are considered as classic biofluids for assessment of systemic biomarkers, saliva is being increasingly viewed as a matrix with a high diagnostic value (141, 145). Saliva collection is economical, safe and can be performed without the assistance of specialized health care personnel, allowing for point-of-injury (POI) sampling. Saliva lacks cellular and soluble components (e.g., coagulation cascade). As the leakage of brain-derived biomarkers in saliva undergoes a process of biological filtration (53, 137, 146), the use of saliva does not require separation steps that are an obstacle to the development of POI blood tests (138, 139). Human saliva is a clear, slightly acidic (pH 6.0–7.0) heterogeneous biofluid composed of water (99%), proteins (0.3%), and inorganic substances (0.2%) (147). Saliva contains enzymes, hormones, antibodies, nucleic acids, antimicrobial constituents, and cytokines (148), which accumulate in salivary glands and are secreted into the oral cavity through acinar cell ducts (149). Available protocols indicate that saliva samples can be stored short term at room temperature and long term at −20°C or −80°C without significant protein degradation, similar to serum or plasma samples (150, 151). Relevant information inherent to the preparation and the technical handling of saliva samples can be found in (150, 152–154).

The whole saliva (WS) proteome, when compared with the plasma proteome, displays a larger proportion (14.5%) of low-molecular-weight proteins (<20 kDa), in contrast to only 7% for the plasma proteome (154). The highest fraction of proteins found in WS ranges from 20 to 40 kDa, whereas the 40–60 kDa range is the largest fraction for plasma. This is consistent with selective permeability between blood and saliva for low-molecular-weight proteins. Five diagnostic alphabets are outlined in saliva, including proteome (153, 155), transcriptome (156, 157), microRNA (158), metabolome (159), and microbiome (160). Saliva is used by clinical laboratories for the detection of secretory IgA antibodies, for the analysis of salivary cortisol and hormones, and for genetic purposes (161–163).

## Salivary Biomarkers of NVU Damage: a New Diagnostic Opportunity?

The salivary proteome has been characterized in CNS disease conditions, such as schizophrenia, bipolar disorders, and genetic disorders including Down's syndrome and Wilson disease (164). An overview of biomarkers identified in saliva for the diagnosis of neurodegenerative diseases such as AD, Parkinson's disease, amyotrophic lateral sclerosis, and multiple sclerosis is provided in (165). Inflammatory biomarkers (e.g., IL-1β, TNF-α, and IL-6) have been quantified in saliva (166).

The deployment of POI salivary tests represents an opportunity for the detection of time-sensitive brain injuries (139–141, 167, 168). NSE was shown as a possible diagnostic salivary biomarker for neuronal damage in patients post stroke (169). Saliva samples have been analyzed for S100B levels, pro-inflammatory factors, and microRNAs in the settings of TBI (168, 170, 171). In particular, S100B levels in saliva were elevated in children post TBI (171). In another pilot study, 15 adult patients with suspected TBI and 15 control subjects were studied. Average salivary S100B level was 3.9-fold higher than blood S100B level, regardless of the presence of pathology [S100B]_saliva_ correlated positively with [S100B]_serum_, and salivary S100B levels were as effective in differentiating TBI patients from control subjects as serum levels (172).

In an attempt to further accentuate the diagnostic significance of salivary testing, we reviewed the literature to obtain potential blood-to-saliva ratios for a number of proteins (Figure 4C). This search was directed to proteins that are not secreted by salivary glands. These proteins can access the salivary fluid by pericellular capillary leak, primarily the crevicular fluid. Importantly, it is currently unknown whether the steady-state permeability of the blood-to-saliva protein diffusion is preserved even at times when the BBB is breached due to brain insults. Literature references were used to examine insulin (173, 174), EGF (175), HGH (19, 176), S100B (18, 54–56, 177–180), adiponectin (181), prostate-specific antigen (PSA) (182), and cytokines (183). To our knowledge, there are no reports of salivary BDNF or NFL levels. All retrieved values were plotted to outline the theoretical cutoff properties of salivary filtration (Figure 4C). Large molecules (e.g., IgG) can be present in saliva owing to active secretion or local production.

Finally, we examined whether blood-to-saliva biomarkers' passage could be empirically predicted or modeled (153). Available data indicate that saliva is not a diluted substitute for the determination of plasma protein levels, as indicated by the incoherent plasma and saliva proteomes (152). Therefore, understanding the kinetic protein passage from blood to saliva is difficult. In the past, a model describing the passage of biomarkers from the brain into the peripheral blood was proposed (27–29). A physiologically based pharmacokinetic model can be used to describe the distribution of drugs and small molecules in body fluids (184). This computational approach can estimate the extent and time course of salivary biomarkers originating from the brain, offering the likelihood of a protein in saliva to be blood-borne (185). The physiologically based pharmacokinetic model used to describe the distribution of brain-derived biomarkers in blood was expanded to include an idealized salivary gland receiving its vascular supply from the external carotid. The venous output was mimicked according to the properties of jugular vein branches. To approximate the combined contribution of transcellular and paracellular pathways of protein extravasation across capillary endothelial cells and salivary gland epithelia, the following equation was used to calculate Js, the transfer of protein from blood to saliva:

(1) $$\begin{array}{r} Js=Jv^{*}\left(1-R\right)*Cp+\left(Cp-Ci\right)*PS \end{array}$$

where Js (mol/min) is the mass transfer from blood to saliva, Jv (ml/min) is the blood flow to the salivary gland, R is the reflectance of the vascular wall, Cp (mol/L) is the concentration of biomarker in the serum, Ci (mol/L) is the concentration of biomarker in the saliva, and P and S refer to permeability (cm/s) and surface of exchange (cm^2^), respectively. The value of reflectance has no dimension and has a range from one (no passage of protein) to 0 (protein passage dictated by diffusion alone). The value of reflectance is derived from pore radius and molecular radius. To estimate PS, we used PS = Jv ^*^ Ci/(Cp – Ci) with salivary flow at 1 ml/min and Ci and Cp at 2.5 and 61.5 mg/ml, respectively. These values were derived by measurements and transfer of albumin levels from blood and saliva. The equation can be greatly simplified by fitting experimental data to confirm their accuracy. Once this is done, the predictors of passage of a given protein are primarily related to its molecular size (vascular wall reflectance) and the presence of a gradient for passage from blood to crevicular fluid. For (1), note that if the reflectance tends toward 1 (large molecular weight), the first term equals zero, thus leaving only the permeability of the capillary wall and the osmotic gradient as variables. Considering that permeability also depends on molecular size, a cutoff for extravasation seems to be mostly related to the size of the permeating protein. By using other computational models, it was shown that the physicochemical properties of proteins were the main predictors of presence in saliva. Among several properties, molecular size was the most relevant (185, 186).

It is important to underscore that the use of saliva samples comes with confounding factors. For instance, gingivitis or periodontal disease can affect the identification and quantification of proteins. It has been shown that submandibular saliva flow rates are lower in AD patients as compared to controls (187), possibly impacting the proportion of proteins detectable (188). In summary, fully defining the qualitative and quantitative characteristics of salivary biomarkers in physiological and neuropathological conditions is important to develop non-invasive point of care applicable to NVU screening.

## Pushing the BBB Limits: Relevance of Peripheral Blood Biomarkers in Human Models of Extreme Brain Physiology

**Figure 5:**
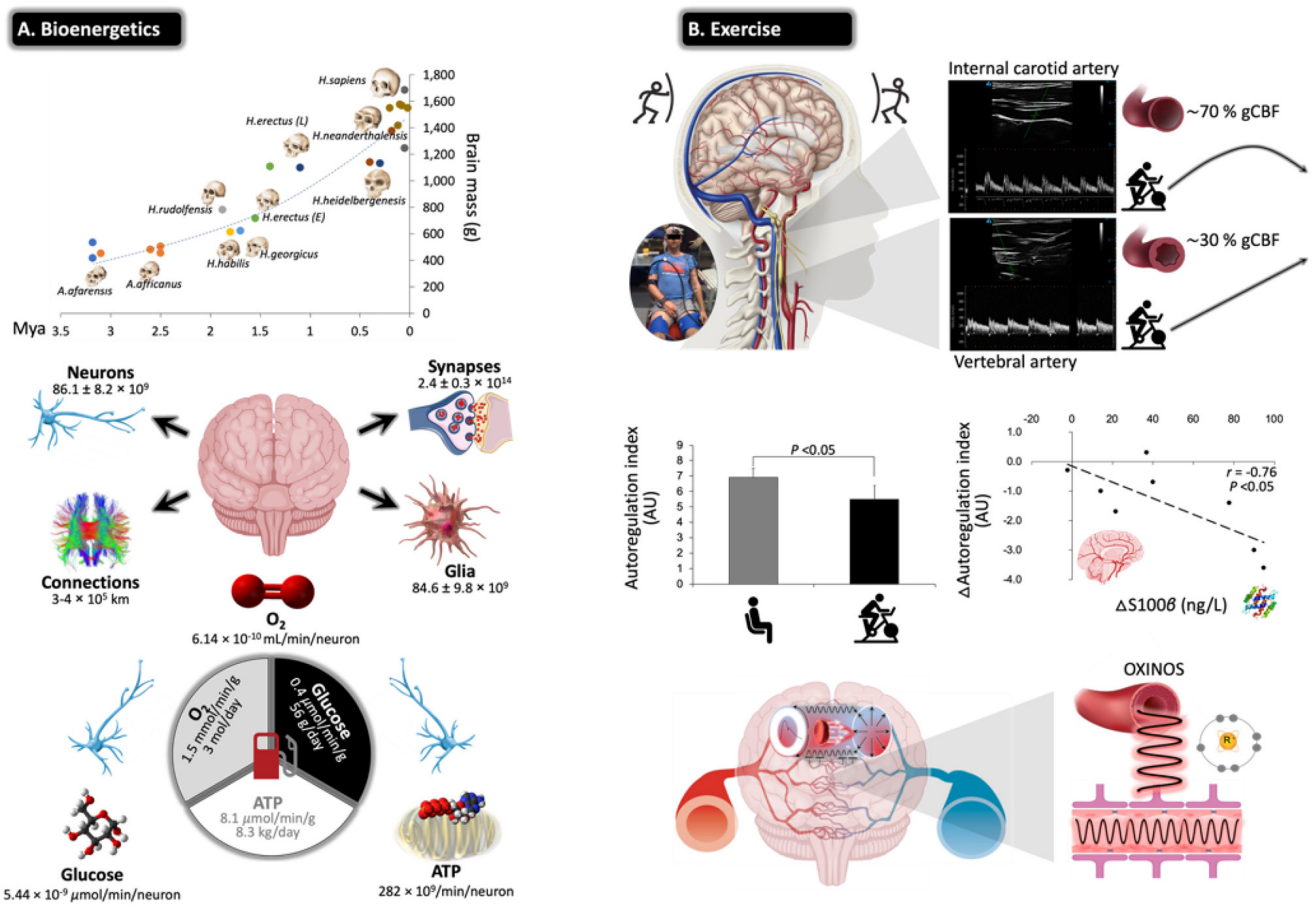
Challenges for the exercising human brain: applicability of NVU biomarkers. **(A)** Evolutionary ‘drive-for-size' with exponential increases in estimated brain mass observed in fossil hominids. Note the structural complexities and corresponding bioenergetic demands that define the ‘modern' human brain, highlighting its limited energy reserves in the form of oxygen (O_2_), glucose, and adenosine triphosphate (ATP) in the face of extraordinarily high rates of neuronal metabolism. This renders the human brain exquisitely sensitive to anoxia and ischemia, and thus, it has developed a sophisticated armory of mechanisms that collectively defend O_2_ homeostasis. Calculations cited and figures modified from (189). **(B)** Physical exercise poses unique challenges for the human brain with perfusion typically characterized by preferential redistribution to the phylogenetically ‘older' regions subserved by the posterior circulation (typical B-mode Doppler images illustrated). This makes teleological sense given that it is one of the most primitive neuroanatomical regions of the human brain, which has remained highly conserved across vertebrate evolution housing (almost exclusively) all the major cardiovascular and respiratory control centers essential for the integrated regulation of autonomic nervous control (190). However, this can come at a cost, with emerging evidence indicating that high flow/pressure and systemic/cerebral formation of free radicals and oxidative inactivation of nitric oxide [oxidative–nitrosative (OXINOS) stress] contribute to impaired cerebral autoregulation and BBB disruption. The latter is confirmed through proportional extravasation of brain-specific proteins, including S100B, in the absence of structural tissue damage. BBB permeability can cause extracellular vasogenic edema resulting in a regional O_2_ diffusion limitation, with the potential to adversely affect cerebral bioenergetics and cognition. This is relevant to patients already suffering from impaired cerebral autoregulation/autonomic dysfunction, including older adults, notwithstanding patients diagnosed with diabetes, hypertension, stroke, and AD (191) (original images by DMB).

Here, we focus on extreme sport settings that can be exploited as ‘human' models to study BBB permeability, neuronal damage, and hemodynamic modifications in a controlled spatiotemporal manner. We review the evidence supporting the use of blood biomarkers to detect neurovascular modifications associated with extremes of cerebral blood flow (Figure 5A). These models share similar pathophysiological features unified by the cerebral formation of free radicals, associated reactive oxygen/nitrogen species (ROS/RNS), and impaired cerebral autoregulation (CA).

## Exercise, Cerebrovascular Regulation, and Blood Biomarkers

Evidence indicates that moderate-intensity continuous training (MICT) and corresponding improvements in cardiorespiratory fitness (CRF) can increase cerebral perfusion and vasoreactivity across the human life span (192, 193), translating into a lower risk of stroke mortality and dementia (194, 195). The primary mechanisms include accelerated neurogenesis, in particular of the hippocampal dentate gyrus (196); reduction in β-amyloid (197); neuro-oxidative inflammatory nitrosative stress (198); proprioceptive adaptations incurred by movements that require sustained mental effort (199); increased brain-derived neurotrophic factor that modulates brain plasticity by promoting neuritic outgrowth and synaptic function (200); and improved BBB integrity and bolstering of tight junctions (201). More recently, high-intensity interval training (HIIT) has emerged as a more time-efficient model of exercise that can potentially promote superior improvements in CRF and cerebrovascular adaptation (191). However, this type of exercise characterized by high-flow/high-arterial-pressure transmission poses unique challenges for the brain with emerging evidence suggesting that an acute bout of HIIT could increase BBB permeability in the absence of neuronal injury (e.g., increased blood S100B and no NSE changes), subsequent to a free radical-mediated impairment in dynamic CA that persists into the recovery period (202) (Figure 5B).

## High-Altitude Mountaineering, Freediving, NVU Dynamics, and Blood Biomarkers

**Figure 6:**
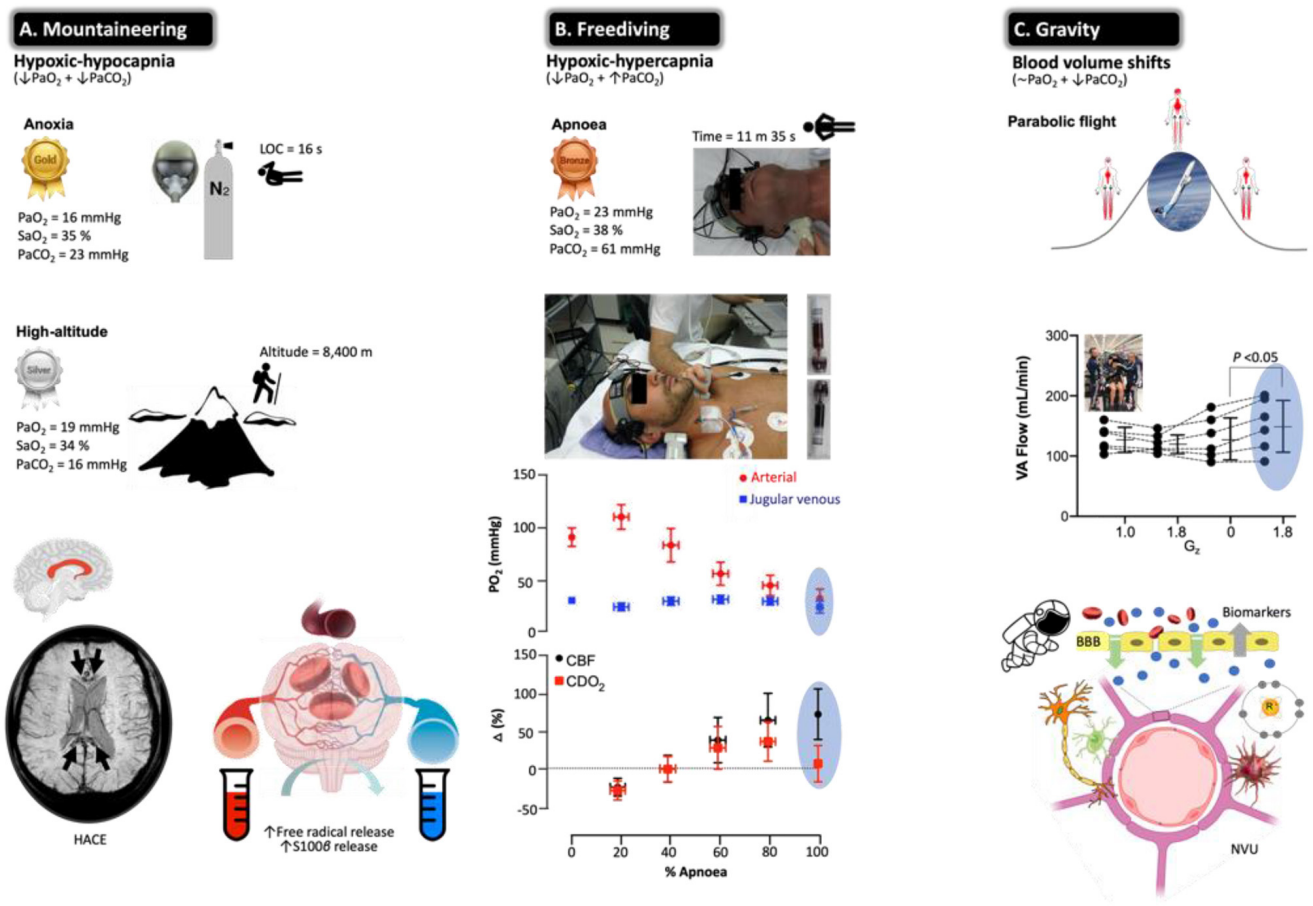
Beyond barriers: the brain under pressure and NVU blood biomarkers. Extreme examples highlighting how the human brain adapts to the most severe swings in circulating oxygen, carbon dioxide, and blood volume recorded in the published literature with data obtained from apparently healthy participants. All examples are unified by a general increase in CBF to ensure preservation of O_2_ and glucose substrate supply. Note the most hypoxemic (notwithstanding hypocapnic/hypercapnic) measurements documented in humans (gold, silver, and bronze awarded according to severity of hypoxemia) who regularly operate at the ‘cusp' of consciousness or indeed beyond. **(A)** Acute exposure to anoxia (pure nitrogen) resulted in an arterial partial pressure of O_2_ (PaO_2_) of 16 mmHg (lowest ever recorded) that resulted in unconsciousness within 16 s (203). The highest recorded femoral arterial puncture performed on an acclimatized mountaineer at 8,400 m on Mt. Everest revealed the second lowest (silver award) PaO_2_ (and lowest PaCO_2_) of 19 mmHg (204). Prolonged exposure to the severe hypoxia of terrestrial HA can result in HACE, a rare albeit deadly syndrome characterized by ataxia resulting in rapid progression to coma. Susceptibility-weighted MRI has revealed hemosiderin deposits (insoluble iron(III) oxide-hydroxide, reflecting micro-hemorrhages) in nonlethal HACE confined to the genu and splenium of the corpus callosum. In combination with vasogenic edematous brain swelling previously documented by MRI studies in healthy participants exposed to acute hypoxia (205, 206) and net trans-cerebral (arterio-jugular venous) outflow of free radicals and S100B (207–209) in the face of impaired dynamic cerebral autoregulation (210) has led to the suggestion that severe hypoxia results in permeability of the BBB subsequent to molecular (free radical-mediated increase in permeability) and hemodynamic (cerebral vasodilatation) stress (211). (**B**) The third lowest PaO_2_ (and highest PaCO_2_) recorded in a single freediver during the course of a static breath-hold (apnea) (212, 213). Note that the level of hypoxemia was so marked there was no observable arterio-jugular venous O_2_ gradient across the brain (marked cyanosis and ‘black blood' visible toward the end of apnea). Hypoxic-hypercapnic increase in CBF was sufficient to offset arterial hypoxemia and preserve the cerebral delivery of O_2_ (CDO_2_) (213), though a mild net trans-cerebral outflow in S100B was observed in a follow-up study taken to reflect mild, diffuse BBB disruption (214). **(C)** Gravitational stress and reciprocal changes in central blood volume were recently shown to increase blood flow to the posterior region of the brain (VA, vertebral arteries) arguably more prone to hyperperfusion injury. This was associated with a free radical-mediated reduction in nitric oxide (NO) bioavailability and mild damage of the NVU reflected by the extravasation of S100B and GFAP (215). Image credits: mountaineering (T*MRI of a 65-year-old woman 7 weeks after having developed HACE at 3,580 m, courtesy of Professor M Knauth, University Hospital Gottingen, Germany); freediver [photographs courtesy of Prof. PN Ainslie and the late Dr. CK Willie, University of British Columbia, Canada (213) with permission]; Gravity (Novespace's ZERO-G Airbus A300 credit: Novespace/CNES/DLR/ESA).

High-altitude (HA) mountaineering (Figure 6A) and freediving (Figure 6B) represent unique physiological models to study severe arterial hypoxemia (O_2_ lack) and hypocapnia/hypercapnia (CO_2_ lack/excess) in ‘extreme' athletes who consistently operate at, or very close to, the limits of human consciousness (189, 212). Diffusion-weighted magnetic resonance imaging has identified increases in brain volume, T_2_ relaxation time (T_2_-rt), and apparent diffusion coefficients (ADCs) in healthy participants acutely exposed to hypoxia, taken to reflect extracellular vasogenic edematous brain swelling (205, 206). These changes were pronounced in the splenium and genu of the corpus callosum, the likely consequence of a unique vascular constitution. Densely packed horizontal fibers characterized by short arterioles that lack adrenergic tone likely render it more susceptible to hyperperfusion edema in the setting of hypoxic cerebral vasodilatation and/or autoregulatory impairment (205, 206). Local sampling of CSF and arterial–jugular venous blood concentration gradients of biomarkers including S100B indicated that BBB disruption is likely minor and linked to increased free radical formation (207, 216).

Some mountaineers, notably those who ascend (too) rapidly to altitudes above 2,500 m and thus not adequately acclimatized, can develop acute mountain sickness (AMS), a primary disorder of the CNS characterized by headache that is associated with, if not the primary trigger for, other vegetative symptoms (217). Traditionally, AMS has been considered a mild form of HA cerebral edema (HACE, the most malignant of all HA illnesses, oftentimes proving fatal) with a common pathophysiology of intracranial hypertension subsequent to vasogenic edematous brain swelling at opposing ends of a clinical continuum. An increase in intracranial pressure (ICP) could potentially result in the mechanical stimulation of pain-sensitive unmyelinated fibers that reside within the trigeminal–vascular system, triggering the symptoms of a headache (218). This makes intuitive sense in light of an early study that identified an increased T_2_ signal in the white matter of mountaineers with moderate to severe AMS in whom clinical HACE had not yet developed (no ataxia or altered consciousness) (219). However, follow-up MRI studies consistently failed to support this concept, with no clear relationships observed between hypoxia-induced increases in brain volume or T_2_-rt and cerebral AMS scores (206, 220). Indeed, the only defining morphological feature that distinguishes the AMS brain from its healthy counterpart is a selective attenuation in the ADC signal taken to reflect intracellular (cytotoxic) edema that likely coexists with extracellular vasogenic edema (206, 220). Attenuation of the ADC signal likely reflects fluid redistribution from within the extracellular space, as intracellular (astrocytic) swelling proceeds without any additional increment in brain volume, edema, or ICP (221). The underlying causes and temporal sequence are unknown, perhaps a reflection of ion pump suppression subsequent to (free radical-mediated) downregulation of Na^+^/K^+^-ATPase activity (211). More recent evidence suggests that a functional impairment in cerebral ‘venous outflow' at the level of the transverse venous sinus may prove the unifying risk factor for AMS (222).

Freediving (Figure 6B) offers yet another remarkable model of severe arterial hypoxemia (189, 212). The static apnea world record currently stands at an impressive 11 min 35 s held by Stéphane Mifsud. However, unlike mountaineers, apnea results in severe hypercapnia, further compounding the cerebral hyperemic stimulus (Figure 6B), with freedivers also having to contend with the additional challenge of elevated hydrostatic pressure when competing ‘at depth' in select disciplines and complications associated with pulmonary barotrauma, nitrogen narcosis, decompression sickness, and high-pressure neurologic syndrome (212). Competitive freedivers oftentimes experience shallow-water blackout due to severe cerebral hypoxia and loss of motor control, clinical signs that are the frustrating cause for disqualification from competition, notwithstanding immunochemical evidence for structural NVU damage, e.g., increased peripheral blood S100B and NSE after a maximal apnea, with potential long-term neuropsychological consequences (223, 224).

More recent, direct approaches have taken advantage of sampling arterial–jugular venous blood and combining regional measurements of CBF during the course of an apnea in champion freedivers (213, 214). Despite no detectable O_2_ gradient across the brain, a truly remarkable observation, CDO_2_ subsequent to increased perfusion was well maintained even at PaO_2_s as low as 23 mmHg (Figure 6B). Similar to the aforementioned acute hypoxia study (207), apnea was associated with a net trans-cerebral outflow of free radicals and S100B (in the absence of any local gradients in NSE or MBP) that may reflect minor BBB permeability due to the combination of hemodynamic (increased intracranial pressure) and molecular (increased free radical formation) stress in the absence of neuronal damage (214). Rather than consider this simply as a damaging maladaptive response, vasogenic edematous brain swelling may prove the adaptive phenotypical response in the hypoxia-tolerant human brain (211, 225).

## Gravitational Stress, Cerebrovascular Regulation, and Blood Biomarkers

Alterations in gravitational fluid pressure gradients caused by the microgravity of orbital spaceflight and hypergravity associated with takeoff and landing pose unique physiological challenges for the astronaut brain. Recent interest has focused on the complex pathophysiology underlying a constellation of debilitating neurological, ophthalmological, and neurovestibular symptoms, known collectively as spaceflight-associated neuro-ocular syndrome (SANS) (226). At the cellular level, microgravity has been associated with a loss of cytoskeletal integrity through dissociation of actin and tubulin bundles (227), and evidence obtained using animal models suggests that BBB disruption may occur during the early phases of unloading induced by suspension or microgravity (228) and during hypergravity induced by prolonged centrifugation (229, 230). In a recent study (215), parabolic flight (PF), a ground-based spaceflight analog, was used as a human model to induce rapidly alternating shifts in central blood volume during repeated exposures to microgravity (0 G_z_) interspersed with hypergravity (1.8 G_z_) (231) to explore how altered CBF impacts the NVU (232) (Figure 6C). Blood flow to the posterior cerebral circulation (vertebral arteries) was selectively elevated during the most marked gravitational differential from microgravity to hypergravity. Posterior hyperperfusion was associated with a free radical-mediated reduction in nitric oxide bioavailability (oxidative–nitrosative stress) and selective increases in blood S100B and GFAP that persisted following return to microgravity, whereas blood biomarkers of neuronal–axonal damage (NSE, NFL, UCH-L1, and tau) remained stable (215). These findings suggest that the cumulative effects of repeated gravitational transitions may promote minor BBB damage due to the combined effects of hemodynamic-molecular stress. While we appreciate that PF is an entirely different stimulus dominated by hypergravity, these findings provide important mechanistic insight to help understand the neurological risks associated with prolonged microgravity during spaceflight, given that increased BBB permeability directly impacts neuronal function, predisposing to neurological sequelae and brain disease (6).

## Blood Biomarkers of NVU Damage: Available Analytical Tools, Limitations, and Controversies

No single ideal peripheral biomarker exists; rather, a suite of biomarkers could have a significant diagnostic impact. In recent years, innovative methods for biomarker detection have been implemented (Table 2). Reaching high sensitivity has several advantages, particularly in the context of neurological settings. Foremost is the ability to detect biomarkers such as NfL, Tau, or GFAP that are readily present in the CSF and in low concentrations in the blood. New technology has enabled the quantification of brain-derived protein biomarkers in blood, getting one step closer to a minimally invasive diagnosis of brain damage and neurodegenerative processes. Furthermore, high-sensitivity methods use microliter quantities of biofluid, allowing the quantification of several analytes and multiplex measurement. As an example, we here provide NfL, GFAP, and tau serum baseline levels as measured in our laboratory using Simoa (Table 2). We include specific LLOD and LLOQ values relative to our particular experience. Obviously, this new technology presents limitations. A shortcoming of high-sensitivity assay resides in the fact that Research Use Only (RUO) kits are not able to provide, yet, a level of robustness and precision that one would expect for a clinical *in vitro* diagnostics (IVD) use. To date, the impact of analytic interference is not sufficiently investigated. Therefore, the expectations formulated following cohort-based studies need confirmations in large preclinical studies and multicentric clinical trials.

**Table 2A:** Available biomarker detection tools.

**Novel diagnostic technology**	**Providers**
Electrochemiluminescent immunoassay	Meso scale discovery
Single-molecule array immunoassay	Simoa® (Quanterix)
Immunomagnetic reduction	MagQu Co
Proximity extension assay	Olink
Immunocapture mass spectrometry	Thermo Fisher, Shimadzu, Agilent, AB Sciex, Waters

**Table 2B:** Examples of analytical parameters.

	**NFL**	**GFAP**	**Tau**
Control baseline levels	8 pg/ml	54 pg/ml	0.35 pg/ml
Limit of detection (LOD)	0.10 pg/ml	0.22 pg/ml	0.02 pg/ml
Limit of quantification (LOQ)	0.24 pg/ml	0.47 pg/ml	0.05 pg/ml

Although the use of blood biomarkers of BBB or neuronal damage is appealing, a number of clinical stumbling blocks currently limit full applicability. The usefulness of blood biomarkers in a given human depends on the availability of reference values, correcting for age, ethnicity, kidney function, and body mass index (29). Adequateness of the blood sampling schedule and availability of baseline controls are crucial for a reliable biomarker outcome. Sample readiness before and after pathological events (e.g., inpatient seizure monitoring and head trauma as in contact sports) provides the optimal framework to calculate biomarker differential in the same individual and within a controlled time frame (24, 25). Availability of *ad hoc* baseline samples (e.g., specific enrollments for sport events and military personnel) represents a robust method enabling personalized medicine.

As examined so far, the appearance of NVU proteins in blood is reported for neurodegenerative diseases (91, 94), brain tumors (115, 117), TBI (25, 233), neurologic manifestations of systemic disease (234), psychiatric diseases, and seizures (21, 53, 235). Peripheral biomarkers have an excellent NPV to rule out disease(s) but have a poor positive predictive value (PPV) to identify a specific pathological condition (27, 28, 46, 53, 236–238). Another concern is the potential contamination related to extra-CNS sources of protein biomarkers. For example, S100B could be derived from adipose tissue with levels directly depending on body mass index (239). A study excluded the impact of adipose tissue on S100B serum levels (23). Elevated serum S100B was reported in patients presenting with extracranial pathology (240), such as polytrauma and burns (66).

Another important question is whether peripheral biomarkers have a prognostic value for the development of long-term brain pathology. Currently, there is no collective agreement on whether an unhealthy BBB may already exist, and could be diagnosed, in an otherwise apparently healthy brain (241, 242). However, recent evidence indicates that subjects presenting early cognitive impairment had preexisting BBB damage. The platelet-derived growth factor receptor beta (PDGFRβ; Table 1) (243, 244) shedding from perivascular pericytes was proposed as a biomarker of BBB integrity anticipating and predicting neurodegeneration (39, 243). A high-sensitivity method for detecting pericyte injury quantifying PDGFRβ in CSF was recently proposed (245). This method could be extended to study brain pericyte–endothelial damage in neurodegenerative disorders. Moreover, repetitive head hits during contact sports [American football (25)] were shown to associate with recurrent BBB permeability and S100B increases in blood. Players experiencing recurrent BBB permeability presented higher serum reactive autoantibodies, with a possible correlation with cognitive defects (25). The clinical significance of repeated BBB damage in sports is currently debated, with evidence pointing to a role in accelerated neurodegeneration (73). Moreover, total tau in blood was reported as a biomarker of axonal damage in hockey (24). Tau and amyloid monitoring in CSF is undergoing validation processes for dementia and AD (246, 247).

## Outlook and Final Remarks

Using peripheral biomarkers to monitor BBB permeability could extend to clinical cases where opening of the BBB is necessary to enhance drug penetration into the brain (13) or when re-establishment of physiological BBB tightness is justified to treat brain diseases (248, 249). Emerging evidence supports a holistic approach to tackle CNS diseases, where neuronal and cerebrovascular contributors of diseases are synchronously targeted. An increasing number of BBB-repairing molecules are currently being tested [for review, see (5, 6)], targeting NVU cells and neuroinflammation. Importantly, BBB biomarker and repairing strategies could become important in the settings of acute or chronic peripheral diseases (infections, metabolic or inflammatory) where immunity and inflammation negatively impact BBB permeability and, consequentially, synaptic transmission (5, 7, 250).

In conclusion, the NVU represents a modern and integrated entry point for the investigations of brain functions, and a continuous technological advancement will be instrumental to improve our ability to link NVU damage with diagnostics. The field of biomarkers of NVU damage, or dysfunction, is expanding together with the use of omic techniques and machine-learning routines for the discovery of signatures of acute or chronic disease conditions.

## Data Availability Statement

The original contributions presented in the study are included in the article/supplementary material, further inquiries can be directed to the corresponding author/s.

## Author Contributions

NM coordinated this effort, generated most of the figures, table, and contributed or wrote all parts. DJ focused on salivary biomarkers and provided parts of figures. DB focused on extreme conditions and biomarkers and providing relevant figures. JB and NM focused on imaging. SL, RO'F, and CH focused on salivary biomarkers and revised applicability of biomarkers. All authors contributed to the article and approved the submitted version.

## Conflict of Interest

DJ was affiliated to the company FloTBI Inc. The remaining authors declare that the research was conducted in the absence of any commercial or financial relationships that could be construed as a potential conflict of interest.
